# Antioxidant and Adaptative Response Mediated by Nrf2 during Physical Exercise

**DOI:** 10.3390/antiox8060196

**Published:** 2019-06-25

**Authors:** Nancy Vargas-Mendoza, Ángel Morales-González, Eduardo Osiris Madrigal-Santillán, Eduardo Madrigal-Bujaidar, Isela Álvarez-González, Luis Fernando García-Melo, Liliana Anguiano-Robledo, Tomás Fregoso-Aguilar, José A. Morales-Gonzalez

**Affiliations:** 1 México Laboratorio de Medicina de Conservación, Escuela Superior de Medicina, Instituto Politécnico Nacional, Plan de San Luis y Díaz Mirón S/N, Col. Casco de Santo Tomás, CP 11340 Alcaldía Miguel Hidalgo, Mexico; nvargasm1900@alumno.ipn.mx (N.V.-M.); eomsmx@yahoo.com.mx (E.O.M.-S.); 2 Escuela Superior de Cómputo, Instituto Politécnico Nacional, Av. Juan de Dios Bátiz s/n esquina Miguel Othón de Mendizabal, Unidad Profesional Adolfo López Mateos, CP 07738 Ciudad de México, Mexico; 3 Escuela Nacional de Ciencias Biológicas, Instituto Politécnico Nacional, “Unidad Profesional A. López Mateos”. Av. WilfridoMassieu. Col., Lindavista, 07738 Ciudad de México, Mexico; emadrigalb@ipn.mx (E.M.-B.); ralvarezg@ipn.mx (I.A.-G.); lgarciam0915@alumno.ipn.mx (L.F.G.-M.); 4 Laboratorio de Farmacología Molecular, Sección de Estudios de Posgrado e Investigación, Escuela Superior de Medicina-Instituto Politécnico Nacional, 11340 Ciudad de México, Mexico; languianor@ipn.mx; 5 Departamento de Fisiología, Laboratorio de Hormonas y Conducta, ENCB Campus Zacatenco, Instituto Politécnico Nacional, 07700 Ciudad de México, Mexico; tfregoso@ipn.mx

**Keywords:** oxidative stress, Nrf2, antioxidants, exercise training, adaptative response

## Abstract

Nuclear factor erythroid 2-related factor 2 (Nrf2) is a powerful nuclear transcription factor that coordinates an antioxidant cytoprotector system complex stimulated by the increase in inoxidative stress (OS). In the present manuscript, we conduct a review on the evidence that shows the effect different modalities of physical exercise exert on the antioxidant metabolic response directed by Nrf2. During physical exercise, the reactive oxygen species (ROS) are increased; therefore, if the endogenous and exogenous antioxidant defenses are unable to control the elevation of ROS, the resulting OS triggers the activation of the transcriptional factor Nrf2 to induce the antioxidant response. On a molecular basis related to physical exercise, hormesis maintenance (exercise preconditioning) and adaptative changes in training are supported by a growing body of evidence, which is important for detailing the health benefits that involve greater resistance to environmental aggressions, better tolerance to constant changes, and increasing the regenerative capacity of the cells in such a way that it may be used as a tool to support the prevention or treatment of diseases. This may have clinical implications for future investigations regarding physical exercise in terms of understanding adaptations in high-performance athletes but also as a therapeutic model in several diseases.

## 1. Introduction

Living organisms have evolved, among other aspects, to defend themselves against environmental aggressions in order the achieve adaptation and ensure survival. In the case of the eukaryotic cells, these have developed a powerful defense system to mediate the damage caused by the production of free radicals generated from cellular metabolic reactions. Reactive oxygen species (ROS) comprise a group of highly reactive molecules derived from oxygen that include hydrogen peroxide (H_2_O_2_) [1], superoxide anion, and the hydroxyl superoxide deriving from organelles such as the mitochondria, the endoplasmic reticulum and peroxisomes or Nicotinamide adenine dinucleotide phosphate oxidase (NADPH oxidases); the mitochondria is the greatest producer of ROS, as a consequence of the electron transport chain coming from complex 1 and 3, where oxygen is converted into superoxide [2]. In addition, the production or reactive species of nitrogen (RSN), such as peroxynitrite coming from nitric oxide, contributes to cellular damage in exercise-induced oxidative stress [3].

The presence of ROS is important for cellular homeostasis in that they intervene as cellular signalers in diverse pathways; ROS are needed for different processes in homeostasis maintenance including cell signaling, cell proliferation and differentiation, adaptation to stress and metabolic adaptation [4]. However, the overproduction of ROS can harm cellular structures such as lipids, proteins, or DNA, increasing the damage due to the increase in oxidative stress (OS) [5]; therefore, maintaining the amount of ROS produced and eliminated is crucial for homeostasis. Thus, the cells have developed sophisticated defense systems that permit cellular adaptation to toxic substances by means of an antioxidant cytoprotector system complex. Nuclear factor erythroid 2-related factor 2 (Nrf2) is a central element of antioxidant regulation in cellular systems and its activation is mediated by OS [6]. When cellular defense capacities are exceeded by insults and the OS cannot be mediated, it is highly probable that pathologies will be generated, such as heart diseases [7], cancer [8], neurodegenerative diseases [9], or accelerated aging [10]. Physical exercise has been demonstrated to activate Nrf2 in response to the increase of ROS, a product of the energy metabolism, including the antioxidant protector response [11]. The objective of the present work is to review how exercise and its diverse forms modulate the expression of Nrf2 and the endogenous antioxidant system thanks to the increase in ROS, the activated signaling systems, and the way they interconnect to generate antioxidant and metabolic adaptative responses to exercise. We also study the epigenetic changes modulated by the training and the benefits with regard to the prevention of alterations in the genome. Investigation models are explored that evaluate the effect of physical exercise under different circumstances in an isolated manner and in synergy with certain bioactive compounds. This attempt contributes part of the support of molecular bases in order to understand the changes generated by exercise that can result in a potential tool in the prevention or treatment of diseases; at the same time, to understanding the molecular mechanisms implied in high-performance (elite) sports that facilitate more efficient training, improving sports performance.

## 2. Nrf2 Activation and Transcription

**Figure 1:**
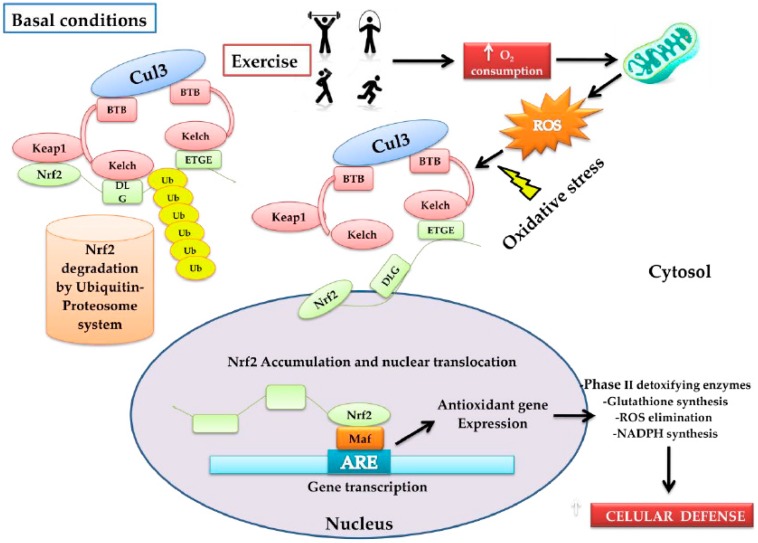
Model that proposes the form in which physical activity activates the nuclear factor erythroid 2-related factor 2 (Nrf2)–Keap1 pathway. Under normal conditions, Nfr2 is found bound to Keap1 through the DLG and EGTE motifs in the Neh2 domain of Nrf2 by the ubiquitin ligase complex Cullin (Cul)3-RING-box protein (Rbx)1 (Cul3). This complex ubiquitinizes Nrf2 for its rapid proteosomal degradation. When physical exercise is carried out, it increases oxygen absorption, transport, and consumption in the entire organism (O_2_ consumption). In this process, the reactive oxygen species (ROS)-producer metabolic pathways are stimulated, increasing oxidative stress (OS). The latter induces oxidation of the cysteine residues present in Keap1, favoring the conformational change of Keap1; consequently, ubiquitination is impeded and Nrf2 dissociates itself from the inhibitor complex. Nrf2 accumulates and translocates into the nucleus, and heterodimerizes with musculoaponeurotic fibrosarcoma proteins MAF proteins, bonding in a specific DNA sequence denoted as the antioxidant response element (ARE), inducing the expression of the antioxidant genes: (a) phase-II antioxidant enzymes; (b) glutathione synthesis; (c) ROS elimination, and d) the synthesis of NADPH, among others. In conjunction with these, an increase in cytoprotector cellular defenses occurs.

The Nuclear factor erythroid 2-related factor 2 (Nrf2) transcription factor is coded in humans by the *NFE2L2* gene, a member of the family of bZIP transcription factors (basic Leucine ZIPper), is known as the antioxidant cytoprotector master regulator, exerting an impact on more than 200 genes associated with the OS response [12]. Under normal cellular conditions, Nrf2 remains inactive through the bonding of its negative regulator Keap1 (Kelch-like ECH-associated protein 1), which is a redox regulator substrate adaptor for the Cullin (Cul)3-RING-box protein (Rbx)1 ubiquitin ligase that directs Nrf2 for its degradation by ubiquitination [6,13]. Keap1 is a cysteine-rich protein that can be oxidized by the presence of ROS and, as a consequence, it changes its conformational state, releasing Nrf2 [6]. Thus, the increase of OS promotes the dissociation of Nrf2 from Keap1, giving rise to its translocation in the nucleus, where it has the capacity to heterodimerize with musculoaponeurotic fibrosarcoma proteins (MAF proteins) to bond with a specific DNA sequence known as the antioxidant response element (ARE, 5′-TGACNNNGC-3′) [14], activating a large group of genes associated with the antioxidant and detoxifying response, such as the phase-II detoxifying enzymes that include Glutathione S-Transferase (GST) and NAD(P)H: Quinone Oxidoreductase-1 (NQO1) [14] (Figure 1).

Nrf2 can return rapidly to its initial form before the oxidative event with a half-life of less than 20 min due to the Keap1 regulator system [15]. However, upon detecting an increase in OS, Nrf2 activation is promoted through modification of the sulfhydride groups of the cysteine residues in the Keap1 binding region, which alters the binding capacity between Nrf2 and Keap1, generating a conformational change [16]. In addition to the 151 residue phosphorylation cysteine processes, a role was described that possesses different enzymes, named stress kinases, on the nuclear localization of Nrf2 phosphorylation, such as the endoplasmic reticulum (ER) stress kinase PERK (PKR-like endoplasmic reticulum kinase) and the energy sensor AMP-activated Protein Kinase (AMPK), which is activated by the increase of ADP, AMP, and creatine during high-performance training due to the energy use of ATP [17,18]. In a similar manner, it was found that Keap1 is not solely relegated to the cytoplasm, it also permits permeation into the nuclear space; in fact, it is known that the Nrf2–Keap1 complex is constantly traveling between the cytoplasm and the nucleus, and as such, the nuclear protein prothymosinα [19] binds to Keap1, facilitating the release of Nrf2 to activate the target genes [15,20]. After the event that generated the increase in OS, the Nuclear Export Signal (NES) facilitates the transport of Nrf2–Keap1 out of the nucleus, ending the antioxidant response signal [21]. Thus, Keap1 can regulate the localization of Nrf2 by means of diverse mechanisms [19]. There are other regulatory mechanisms that have been described that intervene in the binding of the Nrf2–Keap1 complex, for example, p21^Cip1/WAF1^ [22] and BRCA1 [23], which bond with Keap1, and thus avoid being linked with Nrf2. Another mechanism consists of the sequestering of Keap1 by the autophagy adaptor protein p62/SeQueSTosoMe-1 (p62/SQSTM1), permitting the accumulation of Nrf2 [24].

Among the factors involved in the antioxidant adaptative response, Redox effector factor1 (Ref-1) is found, which translocates from the cytoplasm to the mitochondria or to the nucleus in the presence of OS, especially H_2_O_2_ [25,26]. This factor possesses the ability to regulate the transcription of Nrf2 and of other antioxidant genes [27]. Its primordial activity consists of bonding to oxidized transcription factors (Nrf2, Hypoxia Inducible Factor1, and p53); in the case of Nrf2, the latter contains a cysteine amino acid localized in the DNA binding domain (Cys-514) that, in turn, is the Ref-1 binding site for mediating the response to the OS [28]. In this way, Nrf2 remains transcriptionally active for its bonding with ARE in the nucleus, thus activating the antioxidant arsenal [29]. In addition to the presence of ROS, they participate in the activation of the intracellular signaling pathways, such as ERK, MAPK, and PI3K which trigger the Nrf2 pathway [5], this will be discussed further.

## 3. Nrf2, the Antioxidant Response Master Regulator

Nrf2 is known as the antioxidant cytoprotector response master regulator because, upon activation, it coordinates a series of reactions directed toward mediating the OS caused by ROS [3,25]. Nrf2 regulates the glutathione synthesis and metabolism, considering that this element is one of the most important non-enzymatic antioxidants due to its ability to donate electrons, thereby reducing the disulfur links of cysteine amino acids in cytoplasmic proteins [26,27,28]. Furthermore, the expression of the system related to phase II detoxifying enzymes for xenobiotic metabolism is also regulated by this transcriptional factor [28,29,30,31]. On the other hand, NADPH production, which is required for multiple redox reactions and the biotransformation of quinones into less toxic compounds, is also mediated by Nrf2 (Table 1).

## 4. Physical Exercise and Redox Response

### 4.1. Oxidative Stress and Exercise

During the practice of physical exercise, the energy demand of the muscular system increases the consumption of oxygen to a level 10–20 times above that of the rest [32]; this induces the increase in the flow of ROS in the muscle fibers [3]. In terms of bioenergetics, exercise activates the ROS-producing pathways, such as the electron transport chain that, to a certain extent, maintains homeostasis by activating specific basic signaling pathways for the adaptative response to physical training. During exercise, skeletal muscle is the principal producer of ROS, and the cellular sites of greatest production include the mitochondria, xanthine oxidase, nicotinamide, adenine dinucleotide phosphate oxidase (NADPH oxidase), phospholipase A2, and some immune-system cells, such as macrophages, monocytes, neutrophils, and eosinphils [32]. Additionally, it has been reported that the increase in body temperature and the decrease of pH in the blood because of the presence of lactic acid expedites the production of ROS, increasing OS [3]. In addition, it has been proposed that the increase in metmyoglobin deriving from the damaged muscle fibers that interacts with methemoglobin and the peroxides during the training also participates in the generation of ROS [3,32].

In response to the increase of ROS during physical training, especially when it is not exhaustive, the activation of the enzymatic Endogenous Antioxidant System (EAS) is induced, which regulates enzymes such as Glutathione Peroxidase (GPx), Glutathione Reductase (GR), and Catalase (Cat), and the non-enzymatic EAS, which include glutathione and alpha lipoic acid among others. Acute training, as well as chronic training, reveals different responses to OS. Some studies postulate that moderate regular training induces EAS and protects the body from OS-induced damage; in addition to promoting a healthy lifestyle, it reduces the risk of cardiovascular diseases and death. Likewise, in general, an active lifestyle has been related to physiological, metabolic, and transcriptional changes that support the prevention of chronic diseases such as diabetes, as well as preventing or delaying muscle-mass atrophy caused by aging [33]. In another sense, it has been observed that acute exhaustive training dramatically increases the production of free radicals [34]. Vigorous exercise has been demonstrated to occasionally cause sudden cardiac death in sedentary individuals with prior cardiovascular disease, but this is not the case with the habitual practice of moderate exercise [35]. Therefore, establishing a relationship between physical exercise and OS is complex, in that it is necessary to take into account the modality, intensity, and the duration of the training, as well as the individual’s clinical antecedents.

### 4.2. Signaling Pathways and the Epigenetic Changes Induced by ROS during Physical Exercise

Exercise-induced OS exerts an impact on signaling pathways such as Nuclear Factor κB (NFκB) [36] and Mitogen-Activated Protein Kinase (MAPK) [37] that, on being activated, the antioxidant response is induced of the Mitochondrial enzymes Super Oxide Dismutase (MtSOD) and GPx [38], as well as that of Nítric Oxide Synthase (NOS) and greater Catalase (Cat) activity. The role of H_2_O_2_ has been evaluated, not only as an indicator of OS but also as a messenger that transmits signals of redox equilibrium at different cellular sites. Regulation of the redox equilibrium can be affected by means of enzymatic systems or at the transcriptional level [39]. In mammals, H_2_O_2_ modulates the activity of a great variety of transcriptional factors, such as AP-1, Nrf2, CREB, HSF-1, HIF-1, TP53, NF-κB, NOTCH, SP1, and SCREB-1 [40]. In biological systems, the redox equilibrium controls a large variety of basic cellular processes including inflammation, muscle contraction, proliferation, apoptosis, tumor growth, circadian rhythm, and aging, among many others [39,41].

The multiple participation of H_2_O_2_ in all of the events described depends to a great degree on the concentrations reached in the different cellular compartments; on being produced mostly in the mitochondria during the respiratory chain and by the NADH oxidase enzymes, there can be a greater amount in this space, but also in other organelles, such as in the endoplasmic and peroxisome reticulum, where it is produced in important quantities. Under conditions of homeostasis, the concentration of extracellular H_2_O_2_ ranges between 1 μM and0.01 μM intracellularly, where the natural processes of proliferation, angiogenesis, and migration are carried out. The adaptative processes and the response to OS regulated by Nrf2 are performed at extracellular and intracellular concentrations of between 1 and 10 μM and 0.1 and 1 μM, respectively, with the most severe oxidant events, ranging from NFκB-mediated inflammation to tumor growth, fibrogenesis, metastasis, arrest or cell death, achieving concentrations of from 10 up to 1000 μM in extracellular space and of 1–10 μM intracellularly [39]. It is certain that these gradients vary in function type and cellular function, enzymatic activity, cellular compartment, etc. Equilibrium in the metabolic production of H_2_O_2_ functions as a redox marker, for example, on the cytosolic and nuclear peroxyredoxin-1 that regulate the activity of NFκB, similar to the manner in which the Nrf2-Keap1 complex is modulated by the glutathione reductase/glutathione and thioredoxin reductase/thioredoxin systems, an increase in H_2_O_2_ occurs [40]. Moreover, it has been observed that, during the cellular differentiation of myocytyes, an increase of H_2_O_2_ occurs as a consequence of the metabolic demands required by the process that not only functions as a signal, but that also regulates the production of glutathione and the reduced glutathione(GSH)/oxidized glutathione(GSSG) index by activation of the Nrf2-GCL/GR-GSH pathway, exerting an impact on the P13K/Akt/mTOR pathway [42], which will be explained later.

The impact that exercise exerts on the increase in OS is related to the epigenetic changes (47) induced by the antioxidant response system. In themselves, the epigenetic changes are necessary for DNA transcriptional regulation, with methylation being one of the most significant in terms of genetic expression due to its functions in key processes such as genomic imprinting, the repression of repetitive elements, aging, and processes such as carcinogenesis [43]. In this regard, the methylation of DNA promoter regions is associated with the transcription of repression, which can eventually trigger gene silencing [44]. Physical exercise is one of the factors that appears to modify DNA methylation [45,46], generating transcriptional changes in key genes implicated in the response to OS, such as metabolic adaptations and those of the immunitary function, specifically of natural killer cells [47].

Muscle is highly adaptable to the environmental stressors generated in physical exercise. Adaptative response in muscle is controlled by epigenetic changes and methylation is certainly the most explored and DNA transcription Is modulated mostly by this way, limiting the access to transcription machinery by the addition of methyl groups to cytocines followed by guanosines (CpG) in the promoter regions [48]. It has been recognized that the number of genes involved in training adaptation to improve aerobic and resistance capacity, oxidative metabolism, muscle contraction and energy demand. In trained mice, most of the identified genes are related to muscle growth and differentiation such as myogenic regulatory factors (Plexin A2, MyoD and myogenin). The genes that play a part in muscle hypertrophy (Igfbp4) and motor neuron innervations (Dok7) were also found [49]. Hence, DNA methylation is implicated by adaptation to physical training. Interestingly, after acute exercise in a sedentary group of men and women whole methylation DNA decreased, at the same time, a dose-dependent expression of PGC-1a, PDK4, and PPAR-d was observed, suggesting that acute exercise induces gene activation producing DNA hypomethylation on each respective promoter [50]. So, acute or regular exercise induces DNA changes.

Changes caused by acute or regular exercise are observed in antioxidant response methylation genes, such as the Microsomal Glutathione S-Transferase (MGTS1) gene and the neuroprotector OXR1 gene, which exhibit a reduction in methylation. At the same time, the Cat and SOD2 enzymes (18% and 20%, respectively) are significantly greater in the muscular tissue of physically active persons between the ages of 60 and 65 years compared with inactive individuals. Additionally, protein carbonylation is 29% less in active individuals, which indicates greater muscular resistance to damage by OS in physically active persons. Likewise, the repression of methylation is observed due to the effect of the negative regulator of the MAPK p38 and PTPRR pathways, suggesting a reduction of the MAPK p38 pathway in the muscle tissue of inactive individuals [51]. This latter pathway appears to be related to the activation of stem cells in muscle, accelerating the regeneration processes of damaged muscle cells [52]. There is a growing body of evidence that supports the epigenetic modifications related to the training involved in acute as well as chronic physical exercise. In the recent work of Seaborne et al. [53], the authors evaluated the DNA of non-trained individuals at rest followed by acute training in order to later analyze the methyloma of the same individuals submitted to a seven-week chronic Exercise Resistance (ER) Training protocol (three sessions/weekly) to induce muscular hypertrophy), followed by a seven-week rest period, and finally, a return to ER training for seven additional weeks. The result was the identification of a great number of hypomethylated genes (AXIN1, GRIK2, CAMK4, TRAF1) after the training. It was interesting to observe that these genes remained hypomethylated during the rest period, which did not affect the hypomethylated level reached. Additionally, genes were identified (UBR5, RPL35a, HEG1, PLA2G16, SETD3) that participate in hypomethylation and that promote the gene expression linked with the hypertrophy induced by the training. This information had a positive correlation with muscle-mass gain, demonstrating that the increase in methylation, gene expression and the gain in muscle mass were significant, even in the resting period, suggesting an epigenetic memory of these genes. A group of sensitive genes (GRIK2, TRAF1, BICC1, STAG1) was selected after the acute ER training session that remained hypomethylated during the subsequent 22 weeks, with a greater level of gene expression and a gain in fat-free mass in the last training period. The latter speaks to the epigenetic importance possessed by a great number of unexplored genes in different effects, such as hypertrophy and epigenetic memory in skeletal muscle generated by the training [54].

Another observed effect that was most likely related to the previously mentioned epigenetic modifications arising as a response to the increase in ROS in muscle comprises the activation of ubiquitin–proteosome systems for proteic degradation, directed by muscular caspases and calpains [55]. This leads to muscular degradation and consequent atrophy. It is known that repair mechanisms such as the protosomal system for the degradation of damaged proteins undergo a decrease in effectiveness with age [56,57].

Furthermore, exercise-related epigenetic changes extend beyond the individuals who engage in exercise. A study in rats descended from parents that participated in a treadmill-race training protocol, 20 min daily, five days a week, for 22 days prior to mating, had a better spatial development, as well as better DNA global methylation in the hippocampus compared with sedentary rats, suggesting a link between the pre-conceptional physical exercise of their progenitors and the improvement of the cognitive capacity of their descent, possibly related with the epigenetic programming in the hippocampus [58]. For these and other reasons, it is suggested that physical exercise be carried out at all life stages, taking into consideration the capacities or even the limitations of each individual, and that physical exercise should form part of the individual’s lifestyle.

Physical exercise is by far the most effective treatment for the prevention of changes in musculature and the appearance of sarcopenia in individuals of an advanced age, in that it also incites the increase of muscle mass, functional capacity, and mobility, as well as survival [59,60].

### 4.3. Adaptative Responses According to the Exercise Training Modality

**Figure 2:**
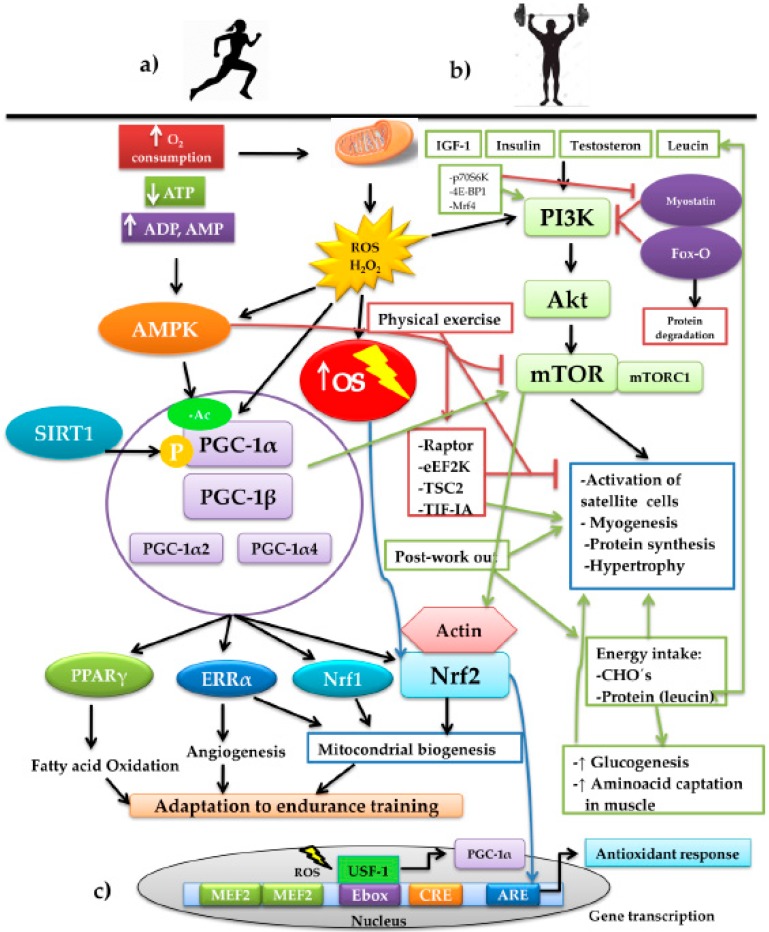
Schematic representation of the interconnection of the signaling pathways involved in the training of aerobic resistance and muscular strength and the adaptations generated by the two training modalities: (**a**) aerobic-resistance sports increase the oxygen consumption (O_2_ consumption). Due to the energy demand, the pathways are activated for the production of ATP, increasing the mitochondrial production of ROS, basically H_2_O_2_, increasing OS, with the consequent decrease of available ATP. The increase of ADP and AMP activate the energy sensor AMPK that, together with SIRT1, activates the PGC-1α. These, upon interacting with other transcription factors, such as PPARγ, ERRα, Nrf1, and Nrf2, induce adaptations to prolonged-duration training through the development of mechanisms, such as the oxidation of fatty acids, the formation of blood vessels, and mitochondrial biogenesis. (**b**) Strength training plus other factors such as IGF-1, insulin, testosterone, and leucine stimulate the anabolizing pathway PI3K/Akt/mTOR. During physical work, this pathway is negatively regulated by AMPK through the mechanisms executed by Raptor, eEF2K, TSC2, and TIF-IA; on finalizing the exercise, the mechanisms are reverted, stimulating protein synthesis and muscular fibrogenesis. The pathway is reinforced by the post-training energy replacement of carbohydrates and the branched-chain amino acid, leucine, which exerts an effect on the activation of satellite cells and myogenesis. We find immersed, in the control of the pathway, Fox-O, a powerful inducer of muscular protein degradation, and myostatin, which negatively regulates the pathway. In counterpart, myostatin possesses the negative regulators p70S6K, 4E-BP1, and Mrf4 that, upon inhibiting it, promote the PI3K/Akt/mTOR pathway. In both exercise modalities, the interaction between the redox-dependent factors, PGC-1α and Nfr2, converges, producing the different adaptations to the training. (**c**) Representation of the nuclear transcription of PGC-1α and the binding Ebox by USF-1 mediated by ROS. On the other hand, activation of the PI3K/Akt/mTOR pathway induces the re-organization of the actin filaments, depolymerizing the actin and forming a complex with Nrf2, which permits its later translocation to the nucleus for its binding to ARE and directing the antioxidant response.

Physical exercise drives diverse adaptative responses to resistance training but also muscular volume if preserving the skeletal-muscle mass is the primary objective, or in any case, creating hypertrophy. Both responses bring together a series of intracellular signaling whose mediation converges in the modulation exercised by the presence of ROS (Figure 2). On considering the effect of the mitochondrial condition during aging, it is crucial to highlight the role of the transcriptional coactivator played by peroxisome proliferator-activated receptor gamma coactivator 1 alpha (PGC-1α), the central element of mitochondrial biogenesis. This molecule of a proteic nature possesses the ability to increase transcription without directly binding to the DNA, given that it interacts with other transcription factors that bind specific sequences, inducing the genomic expression associated with mitochondrial biogenesis, angiogenesis, and the increase of fatty acid oxidation. Thus, PGC-1α is considered a key piece in promoting adaptative responses in endurance training [61,62] (Figure 2a). PGC-1α interacts with other transcription factors to coordinate different programs in response to OS. PGC-1α interacts with ERRα, Nrf1, and Nrf2 in mitochondrial biogenesis [63,64]. Upon interacting with myocyte enhancer factor-2 (MEF2), it stimulates the synthesis of myosin heavy chains of type IIX fibers, forming oxidative fibers, the so-called “fast-twitch fibers”, in skeletal muscle (SkM) [65]; fast twitch fibers are still oxidative fast-twitch thus would most likely impact endurance based activity. Additionally, with peroxisome proliferator-activated receptors γ (PPARγ), it promotes the oxidation of fatty acids [66] and, upon interacting with the estrogen-related receptor α (ERRα), it induces the formation of blood vessels [67]. PGC-1α is regulated by several factors, one of which is the increase of synthesized ROS when aerobic metabolism is carried out, especially in the presence of H_2_O_2_. Upon prolonging the training time, for example, in aerobic-resistance sports, the production of ROS increases and with it, OS. Therefore, EAS is activated, as mentioned previously; moreover, it has been recognized that part of the ROS produced activates the transcription of the alternative promoter PGC-1α2, expressed to the greatest degree in skeletal muscle [67] through the modulation of the upstream stimulatory factor 1 (USF-1). The latter binds to an enhancer box (Ebox), which is a gene promoter region charged with its transcription, together with another two promoter regions: cAPM Response Element (CRE) and MEF2 [68]. The binding of USF-1 to Ebox in the first 850 base pairs of the promoter PGC-1α2 is dependent on the presence of ROS [69] (Figure 2c).

In another respect, during high-intensity sports, energy expenditure increases, together with the depletion of ATP, with the subsequent increase of ADP, AMP, and creatine. ADP and AMP bind to the AMP-activated protein kinase (AMPK) protein, activating it allosterically, protecting it from dephospholylation [70,71]. It is noteworthy that there are two pathways for activating PGC-1α: phosphorylation and acetylation. The former becomes more active to PGC-1α; thus, it is convenient to maintain PGC-1α that is more phosphorylated and less acetylated [61], while phosphorylated AMPK can modify the charge of PGC-1α by the addition of the negatively charged phosphate groups and can regulate their transcription through MEF2 [72]. Deacetylation is carried out by the family of Sirtuin (SIRT) deacetylases; specifically, SIRT1 is responsible for removing acetyl groups to PGC-1α, promoting its activation, while, in turn, SIRT1 is activated by caloric restriction [73]. In this regard, exercise plays a crucial role when carried out under fasting conditions or insufficient-ingestion conditions of foods that sustain energy support [61].

Meanwhile, PGC-1α and Nrf2 possess a close relationship to the modular pathway together with elements of the antioxidant response. Although the direct molecular pathway has not been elucidated, it has been speculated that the pathway of one of these two transcription factors can be influenced by the other, probably because both are dependent on ROS. Additionally, Nrf2 regulates various mitochondrial enzymes that are also related to the activation of PGC-1α [74]. In mitochondrial biogenesis, Nrf2 not only acts with PGC-1α but also with other transcription factors, such as nuclear respiratory factors 1 and 2 and mitochondrial transcription factor A (MTFA), which encode proteins of the mitochondrial complexes and active enzymes in the mitochondrial matrix [75,76]. Under proliferative and growth processes, Nrf2 is immersed through the regulation of the pentose phosphate pathway with the expression of the enzyme phosphorybosyl pyrophosphate aminotransferase (PPAT) and that of methylenetetrahydrofolate deshydrogenase 2 (MTHFD2) for the biosynthesis of purines and nucleotides [77]. Mitochondrial quality control is influenced in the same manner by Nrf2 by inducing the process by which dysfunctional mitochondria are selectively phagocyted by autophagosomes and dispatched to the lysosomes for their degradation and recycling, a process known as mitophagy (95), which permits the maintenance of homeostasis and mitochondrial integrity [74].

Whatever the training modality, the practice induces the expression and activation of PGC-1α that, in addition to modulating the adaptative responses in the exercise, has been associated with diminution of the apoptosis of muscle cells [78,79], reduction of inflammation, greater capacity of the cellular systems to tolerate OS, and activation of the proteosomal system [59,80]. In view of the latter, one should consider that the benefits of practicing some physical-training modality are not only for elite athletes; any individual can prevent or delay cellular aging [59].

Parallel to these effects, one of the inherent effects of exercise is the maintenance or increase of muscular volume and strength [81], dependent on the equilibrium between anabolic and catabolic reactions, in which physical exercise and the availability of energy plays a preponderant role [82]. Disequilibrium in protein/energy consumption plus the increase in protein/muscular degradation will condition the catabolism of the fat-free mass [83]. Physical exercise, above all that of strength, in itself stimulates the anabolic pathways, the most important of which is the phosphatidylinositol 3-kinase (PI3K)/serine threonine kinase (Akt) pathway. This pathway stimulates the target of rampamycin pathway in mammals (mTOR), known as thePI3K/Akt/mTOR pathway, responsible for the synthesis of proteins and muscle fibers [84] (Figure 2b). This pathway is modulated by factors such as insulin-like growth factor 1 (IGF-1) [85,86], insulin [87], testosterone [88], the essential branched-chain amino-acid leucine [89] and, of course, exercise [90]. The latter has also been related to the activation of satellite cells, promoting the synthesis of myofibillar proteins [91]. In cardiac tissue, it has been found that PI3K (p110a) is the main mediator of non-pathological physiological hypertrophic growth under conditions of physical training [92]. The PI3K/Akt pathway responds to signals emitted by growth factors that induce proliferation and cellular growth in muscle and cardiac tissues in athletes [93]. The activity of the insulin receptor (IR) stimulated by insulin and IGF-1 plays a determinant role in the activation of this anabolizing pathway. It has been reported that the ablation of IR in mice generates respiratory-chain dysfunction and low ATP production in the mitochondria [94]. Under the effect of ROS induced by exercise, the PI3K/Akt pathway is stimulated, and OS produces a re-organization of the actin microfilaments, causing depolymerization, which in turn, allows the Nrf2 to form a complex with actin to later translocate it to the nucleus [95]. Additionally, the existence of a complex of isoforms of the insulinic growth factors has been described that, taken together, are denoted asthe mechano growth factor (MGF). These are activated with mechanical work and, together, appear to promote muscle recuperation and repair, initiating the hypertrophic response through the fusion of the satellite cells with the existing fibers, ensuring cellular growth [10,96,97].

Similarly, the mTOR pathway and protein synthesis are inhibited by the AMPK pathway blocking the mTORC1 (mechanistic target of rapamycin complex 1), responsible for connecting factors of growth and anabolizing stimuli with the cellular-growth pathway [17]. AMPK can block the pathway in different ways: (1) phosphorylation and inactivation of the regulatory-associated protein of mTOR (Raptor) [98]; (2) phosphorylation and activation of the negative regulator of mTORC1 tuberous sclerosis complex 2 (TSC2); (3) activation and phosphorylation of the eukaryotic elongation factor 2 kinase (eEF2K), which inhibits the activity of eukaryotic elongation factor 2 (eEF2), an indispensable element for protein synthesis in skeletal muscle, an element that has shown to be diminished during physical resistance exercise [99]; (4) phosphorylation and inhibition of transcription initiation factor IA (TIF-IA), a transcription factor of RNA polymerase I, which inhibits the synthesis of ribosomal RNA, limiting the synthesis of proteins [100]. During the execution of exercise, these mechanisms are activated; however, on finalizing the physical work, they are reverted, increasing the synthesis of proteins in response to the increase in the sensitivity of insulin, the greater uptake of glucose, the reuptake of muscular amino acids, and post-training glucogenesis [101]. This is positively reinforced by the timely contribution of food-derived protein and carbohydrates upon terminating exercise [102].

In a recent study [103], it was reported that interval running training inhibited the expression of inflammatory molecules and reduced the OS, repressing the MAPK pathway. It stimulated muscle-mass gain in ovarietomized rats through the promotion of muscle-growth factors such as myogenin, the phosphomechanistic target of rapamycin (p-mTOR), SIRTruines (SIRT), MyoD, and bone morphogenic proteins (BMP). In addition, a reduction was registered in bone reabsorption as well as in the promotion of osteogenic differentiation and in the formation of bone mass by modulation of the bone-marrow macrophages. Therefore, it is suggested that this training type can be implemented for the prevention of muscular wasting associated with aging. In counterpart, myostatin and the forkhead box transcription factor O (Fox-O) [104], a powerful inducer of the ubiquitin–proteosome system for muscle fibers degradation [105], together these appear to be implicated in the interruption of the mTOR pathway. Myostatin negatively controls muscular growth [106]; therefore, the repression of the myostatin signaling pathway inhibits the transcription of genes implicated in protein synthesis, stimulating muscular anabolism by means of the regulation of the Akt/mTOR pathway [90,107,108]. The transcriptional modulation of components of the Akt/mTOR pathway (Akt, p70S6K, and 4E-BP1), as well as of the myogenic regulator factor Mrf4, are involved in the increase of the musculoskeletal mass induced by the suppression of myostatin [109]. The apparent enlargement of muscle-cell size is due, in part, to the phenomenon of “cell swelling”, due to the osmotic changes, this event is experienced by the rapid contraction fibers. In this regard, type IIA and type IIB are better adapted, expressing a greater number of aquaporins [110]. Cellular enlargement is an indicator of protein synthesis, diminution of proteolysis, and myofibillary growth; this latter phenomenon is crucial in muscular remodeling in response to strength training [10].

Simultaneously, the activation of PGC-1α4 also promotes the synthesis of IGF-1, intervening in the formation of muscle mass [107]. Recently, the activities of PGC-1α and PGC-1β were described, they are positive regulators that exert an influence on protein synthesis and on muscle-fiber diameter through the Akt/mTOR pathway in a model of physical exercise in humans [111]. Due to this, it is noteworthy that engaging in physical exercise involves a great variety of signaling pathways and transcriptional processes that, taken together, induce the adaptative changes that, in turn, are related in some way with the production of ROS. Due to this, it is worthwhile to note the effects that normal physiological levels of ROS can trigger in adaptative responses to physical exercise, contrary to what continues to be considered concerning OS in terms of cellular damage and compromised in sports performance. The consumption of vitamin or dietary supplements has become a very common practice among athletes, involving exorbitant amounts of bioactive compounds, which in effect, have exhibited antioxidant and protector activity, but that can block the adaptative physiological responses, such as the antioxidant endogenous response, mitochondrial biogenesis, natural defense mechanisms, and insulin sensitivity [112]. In other words, hormesis (exercise preconditioning) can be observed as being affected by the inopportune consumption of antioxidant supplements. This does not mean to say that this consumption is harmful but, instead, that the consumption of these products should fall within the jurisdiction of a health and nutrition expert after individual evaluation of the conditions and requirements of each athlete [113,114].

## 5. Regulation of Nrf2 by Physical Training

There is increasingly more information that supports the role that Nrf2 plays in the response to OS and to the physiological adaptations observed during physical training. The practice of exercise is a potent stimulus that promotes physiological and metabolic adaptations that permit confronting the periodic increase of the burdens of physical work, such as changes in plasticity and cellular remodeling. Such adaptations are, in turn, the result of a complex relationship between regulator factors and signaling processes [115]. The OS generated by physical exercise induces the endogenous antioxidant defense systems that themselves, to a great extent, are regulated by Nrf2. The effect exerted by the practice of PE on the activation and possible regulation of the EAS interact to mediate the oxidation of cellular structures such as lipids, proteins, and nucleic acids [11,116] (Table 2).

### 5.1. Nrf2 in Aerobic Exercise Models

The study conducted by Wanget et al. [121] evaluated the effect exerted by acute exercise on the signaling pathways to activate the antioxidant response in skeletal muscle in ICR/CD-1 mice. The principal finding of the authors was that acute exercise induced an increase in the expression of the genes Ref1 and Nrf2, as well as the production of the proteins Ref1 and Nrf2, EAS enzymes CuZnSOD and MnSOD, and the content of glutathione. In other words, acute exercise promotes the production of H_2_O_2_, inducing the activation of the Ref1/Nrf2 pathways. Additionally, an increase in Nrf2 expression was positively correlated with the increase of GSH in skeletal muscle, indicating that elevation of OS stimulates the synthesis of the glutathione. This effect of physical exercise allows for higher deposits of reduced glutathione (GSH) in muscle [126]. This is contrary to what has been observed in the myocardium where, to our knowledge, an increase has not been reported in the concentration of GSH post-training [125]. In addition, the expression of Nrf2 increased the activity of MnSOD; however, this was not the case in CuZnSOD. This could be due to the manner in which SOD isoforms are found distributed intracellularly. This can affect the way that they act under different physiological conditions of stress [127]. Previously, these same authors had obtained data on the expression of p66shc forkhead box O3a (Fox-O) and the modulation of Cat and total SOD on increasing the concentration of H_2_O_2_ by the effect of acute physical exercise [128].

One of the most notable phenotypical adaptations as a response to physical training is the increase in content or structure or mitochondrial biogenesis. Such a process depends on the inter-relationship of various transcription factors such as PPARα, EERα, and Sp1, as well as by members of the PGC1 family (PGC1-αand PGC1-β). In contrast, the role of Nrf2 in mitochondrial biogenesis is not yet clear; thus, in a study, the role played by Nrf2 in skeletal muscle and its impact on the mitochondrial content, as well as the relationship with performance during exercise in mice aged three and 12 months, was studied [117]. Both mice, that is, Nrf2 Wild-Type (WT) and Nrf2 Knock Out (KO), were submitted to a training protocol of voluntary wheel running to determine whether Nrf2 was required to induce mitochondrial biogenesis in skeletal muscle. The results suggest that Nrf2is required for basic mitochondrial function because the silencing of Nrf2 severely affected the mitochondrial respiration of the intramyocellularf raction (IMF) in 40% of the muscle samples of KO mice, while it significantly increased the emission of ROS during the process. Significant differences were not observed in the composition of IMF mitochondria between the WT and KO groups, suggesting that the probable mitochondrial changes and the production of ROS may be due to the differences in the expression of uncoupling protein 3 (UCP3), in that it has been demonstrated that the content of this protein is 1.3 times greater in the IMF compared with the fraction of the mitochondrial subsarcolemma (SS). UCP3 promotes the antioxidant response of Nrf2 because, under conditions of stress due to ROS, UCP3 increases proton conductance of the internal mitochondrial membrane, diminishing the creation of superoxide [129]. A significant difference was not observed between aerobic capacity and fatigue in young (three months of age) and old mice (12 months age), which can be due to the fact that there was no alteration of mitochondrial respiration in both genotypes. There was probably not an insufficient supply of ATP flow; thus, this did not affect aerobic resistance. On the other hand, while in maximal muscular strength there were no significant differences, the aerobic resistance of Nrf2 NO mice was observed as being reduced during an isolated muscular test. The highest degree of fatigue in KO animals may be associated with the elevated level of exposure to ROS, a fact proven on noting an increased level of ROS in the mitochondrial samples of these animals. It is speculated that the high level of ROS, partially the result of the mitochondrial dysfunction, can promote alterations in the structure and function of these muscular filaments [130], diminishing sensitivity to calcium, favoring a higher level of fatigue. A relevant point was that, on evaluating the importance of Nrf2 in mitochondrial content, a significant difference was not observed between both genotypes, in animals aged three, as well as in those aged 12 months. However, the activity of Cytochrome C Oxidase (COX) and of the COXIV subunit increased, induced by the training of the WT animals, suggesting that the nuclear translocation and signaling induced by Nrf2 can be crucial for increasing the activity of this enzyme in the electron transport chain. Nrf2 is found to be essential for the stoichiometric adaptations of the proteins in response to the training, but not for the functioning of the remaining organelles. Taken together with the latter, the results indicated a clear reduction of NQO1 in KO animals, which can affect them to metabolic alterations. Therefore, it is suggested that the transcriptional activity of Nrf2 induced by acute exercise induces adaptations in the mitochondrial proteins. In contrast, chronic exercise leads to a reduction of Nrf2 in WT animals, in addition to reducing defects in the mitochondrial function due to the absence of Nrf2. Thus, the power of physical training to activate a set of physiological adaptations that benefit muscular function beyond a sole signaling pathway is highlighted.

Similar results were found in the study conducted to evaluate the effect of stress due to acute exercise on the activation of Nrf2/ARE in cardiac muscle in mouse [125]. The results tested the positive relationship between ROS production and acute exercise, activating Nrf2/ARE, consequently, the antioxidant program (Cat, Gclm, Nqo1, Gclc, Gpx1, G6pdx, Gsr, Nox, SOD, and GSH/GSSG) in WT mice in comparison with disruption of the pathway in mice that underwent silencing of the Nrf2^−/−^ gene. Additionally, Nrf2-/animals exhibited a significant increase in ROS levels; therefore, an incapacity for inducing the response of the antioxidant cytoprotector system mediated by Nrf2/ARE. This sustains the practice of physical exercise as a possible non-pharmacological therapeutic agent for cardiac conditions, transcriptionally and translationally, giving rise to the Nrf2-mediated antioxidant system. Age, volume, and intensity play a crucial role in the antioxidant response. Another investigation performed in old mice proved that high-intensity exercise renders the cardiac muscle susceptible to elevated OS levels; in contrast, moderate-intensity exercise promotes the activity of Nrf2 and of the antioxidant cytoprotector system in terms of damage due to OS [34].

It is interesting to observe different responses to genetic conditions or those of age and training protocols in the studies reported. For example, under conditions of OS and age, suppression of Nrf2 induces apoptosis and diminution in the capacity of muscular regeneration; however, acute physical exercise exerts an effect on the activation of compensatory mechanisms due to the loss of Nrf2, such as the independent activation of antioxidant systems coordinated by other sensors such as PGC1-α. In old mice that underwent Nrf2 disruption, a diminution was reported of the population of stem cells (PAX7) and a reduced cellular differentiation due to the low expression of MyoD, with the simultaneous activation of the degradative pathways of ubiquitin and apoptosis, compared with WT animals after one week of recuperation in an acute physical-exercise protocol [131]. Responses to OS, to a great extent, are intervened by age and the physiological changes that naturally accompany it. For example, the response to a dose of acute training is different in rats aged 28 months and those aged five months. The stress–response proteins (proteasomes, Hsp70, SOD1, HSF-1, and NF-κB) considerably increase upon evaluating the Akt and Erk pathways and the expression of PGC-1α, Nrf1, and TFAM [132]; in other words, the capacity to respond to the increase of OS due to training is less when it is not practiced habitually. In contrast, when exercise forms part of the lifestyle, there is a greater ability to respond to stress, inducing antioxidant systems more efficiently [10]. In individuals aged 71 years, an increase was observed in mitochondrial biogenesis after acute training at 75% of VO_2max_; however, the response is not the same as in young adults, suggesting that the maximal homeostatic adaptative response is altered with age [133].

In humans, studies on Nrf2 are limited. In 2016, Done et al. [116] reported, after a 30-min cycling test, a 70% VO_2max_ in young males (23 ± 1years of age; *n* = 10) and in males of an advanced age (63 ± 1 years; *n* = 10); the cellular level of Nrf2 increased significantly between both groups, but the nuclear content was only significant in the young males, as was the expression of enzymes HO-1 and NQO1; the expression of proteins HO-1 and NQO1 was similar in both. This suggests that a training session can increase the cellular levels of Nrf2in both young and adult males; despite this, nuclear translocation and expression of the antioxidant system cannot be ensured, probably due to the biological changes associated with age. On the other hand, these same authors, one year later, reported another study [119] in which they compared the effect of high-intensity interval training (HIIT) vs. moderate exercise training (MET) at Nrf2 levels in young adults (25 ± 1 years of age; *n* = 16). Before and after the HIIT session (30 min), blood samples were taken to estimate OS markers, age, and other conditions, but this was not so in the entire cell. OS indicators in plasma, 8-isoprostanes, and peripheral-mononuclear-cell glutathione reductase (GR) increased in response to the training; in contrast, SOD exhibited no significant modifications. This supports the previously mentioned conclusions that the responses vary according to the training modality, training duration, age, and other conditions.

The recovery from physical exercise under hypoxic conditions can alter the adaptative response to oxidative stress, independently of the activity. A significant increase of NFE2L2 expression around 20% accompanying SOD2 elevation in 42% in normoxic conditions was reported; however, an hypoxic environment alters the response to oxidative stress and metabolic adaptation [134].

### 5.2. Nfr2 in Resistance Exercise Models

Studies regarding resistance to exercise models are quite limited in number; however, a study has been carried out on patients with chronic kidney disease (CDK) and hemodialysis in which they were subjected to a muscle resistance training program (RTP). The aim of the study was to evaluate the effect of RTP on Nrf2 and NF-κB expression. The RTP consisted of three sessions per week on the same day as the hemodyalisis. Peripheral blood mononuclear cells were taken three days before and after training. The results showed an increase in Nrf2 expression and GPx activity after training compared with the sedentary control group. This suggests that resistance training could improve the protective and antioxidant response in CDK patients undergoing hemodyalisis [135].

An investigation attempted to prove three different RTP with four Wistar rat groups divided into: (1) Untrained (UT), (2) muscular resistance training (RT), (3) hypertrophy training (HT), and (4) strength training (ST). After 12 weeks of training on alternative days, an increase in the antioxidant response indicators including SOD, Cat, and GPx activity was reported, with the latter being significantly higher in the HT group compared with the UT control. The highest level of OS was reported in the HT group, the Thiobarbituric acid reactive species (TBARS), thiol and carbonyl compared with the control group. These results suggest that the increase of induced-oxidative stress by the hypertrophy training triggers the antioxidant response as a defense mechanism [136].

### 5.3. Physical Exercise and Bioactivador Compounds of Nrf2

A concept that has been coined is hormesis, which makes reference to the fact that cellular systems require a moderate amount of ROS, which increases the OS in order to drive the endogenous antioxidant defenses. In this manner, the cell will be capable of supporting insults by OS more efficiently in the future [137] and the mitochondria play a fundamental role in this process (mitohormesis) [138]. In this respect, Nrf2 can be considered as being an indispensable element on regulating a good part of mitochondrial physiology, such as biogenesis and integrity, the oxidation of fatty acids, cellular respiration, and the synthesis of ATP [139]. Induction of the antioxidant system by exercise has been sustained in various ways, making reference to the fact that the response depends on the intensity, volume, and frequency of the exercise, among other elements. ROS production during exercise has proven to serve as a triggering factor for the activation of molecules that serve as cellular messengers that activate redox-sensitive signaling pathways in the adaptative response, maintaining hormesis [140]. In some manner, the constant exposure to stress generated by exercise stimulates the cellular protector systems, preparing the organism for future mechanical, oxidative, or metabolic insults that, under other circumstances, would lead to diseases. In this regard, the term “hormesis” under training conditions is denoted as preconditioning exercise (PCE). It is responsible for producing transcription factors, stress–response proteins, antioxidant enzymes, mitochondrial biogenesis, and adaptor proteins, which in general, renders the cellular systems more resistant to damage and diseases. The study of PCE can provide valuable information for understanding cellular defense mechanisms and survival with the purpose of preventing diseases such as ischemia, diabetes, cancer, or even delaying aging [141].

In several studies involving models of regular exercise of moderate intensity, whether acute or chronic, the benefits are clearly manifested by the expression of Nrf2 and the antioxidant pathway in different organs and systems; however, when exercise training volume is exhaustive, the possible protective effect can be retained, even more so, could favors the increase of OS, compromising cellular homeostasis [34]. Therefore, a window of opportunity has arisen to propose the timely administration of the bioactive compounds of foods or plants that can drive the expression of Nrf2 and of the entire antioxidant response element (ARE). The work elaborated by Oh et al. [118], in which the authors evaluated the effect of the intraperitoneal administration of sulforaphane (SFN) in wild-type male mice (Nrf2^+^/^+^) and Nrf2-null (Nrf2^−/−^) in mice aged 11–13 weeks that were submitted to an exhaustive training test (treadmill test). The results revealed that the distance run byNrf2^+^/^+^ mice injected with SFN was significantly greater than that of mice who did not receive the supplementation. In the markers of oxidative damage, TBARS were significantly lower 18 h after exhaustive exercise in Nrf2^+^/^+^ mice injected with SFN than in Nrf2^−/−^, and even with Nrf2^+/+^ that were not administered SFN. The glutathione oxidized/reduced index (GSSG/GSH) was significantly lower in Nrf2^−/−^ than in the other groups. The markers of muscular damage, such as creatine phosphokinase (CPK) and lactate dehydrogenase (LDH), were significantly lower in Nrf2^+^/^+^ mice compared with the other groups. In contrast, a significant elevation was not observed in the lactate levels in the blood of Nrf2^+^/^+^ mice, while the authors did report an increase in the levels of the remaining groups. These data suggest that the administration of SFN can exert a protector effect in muscle under conditions of exhaustive exercise. The analysis of mitochondrial biogenesis demonstrated a significant increase of AMPKα in the Nrf2^+^/^+^ group; however, there were no significant differences in the expression of SIRT1 and PGC1α in any of the groups. An increase was observed in the level of copies of mitochondrial DNA (mtDNA) with respect to the copies of nuclear DNA (nDNA), which is considered a good marker of biogenesis in Nrf2^+^/^+^ mice that received treatment with SFN. On the other hand, there were no markers of the RNAm expression of NRF-1, TFAM, p53r2, COXIV, SCO1, and SCO2. These data suggest that there was a weak connection between the activation of mitochondrial biogenesis and the administration of SFN. Additionally, there was significant expression of the genes that regulate Nrf2, including OH-1, NQO1, γ-GCS, and Cat in the muscular tissue of gastrocnemius and soleus of Nrf2^+^/^+^ mice treated with SFN. This investigation suggests that supplementation with SFN induces SFN regulation and that the antioxidant response can play an important role in attenuating fatigue and in reducing the OS caused by exhaustive exercise, suggesting an improvement in the capacity of resistance to training. Curcumin has also demonstrated improvement in the response to training in mice with cardiac failure by inducing the antioxidant response mediated by the activation of Nrf2 [142]. In this study, the conditions were supplementation with curcumin at a dose of 50 mg/kg/day for eight weeks and under a treadmill exercise test at 15° ofinclination, startingat a speed of 6 m/min for 6 min, followed by an increase of 3 m/min every 3 min to exhaustion, prior to the test a conditioning training was conducted for three days for 20 min/day. The results indicated that the mice that received supplementation with curcumin presented better tolerance to exercise: the samples of the soleus and extensor digitorum longus (EDL) muscles presented fewer indicators of fatigue, and the expression of antioxidant enzymes HO-1 and SOD2 increased. In addition, the myogenin and MioD elements that intervene in the formation of muscle cells were higher, with a reduction of ubiquitination markers MURF1 and atrogen-1.

Therefore, the use of bioactive products also comprises an opportunity to promote the activation and effect of Nrf2 in the antioxidant response under situations of OS caused by exhaustive training or even by pathological conditions where Nrf2 protection properties could be limited.

## 6. Nrf2 and Modulation of Energy Metabolism during Exercise

In addition to the antioxidant system complex, exercise activates signaling pathways that regulate gene transcription for the production of proteins involved in mitochondrial function and energy metabolism [143]. The regulation of several of the detoxification pathways mediated by Nrf2 is based on the constant supply of glucose. This supports the idea that Nrf2 not only participates in the regulation of those pathways but also supplies the metabolites participating in them. The homeostasis of glucose is essential for the maintenance of biological functions and of the energy metabolism of the SkM and of the whole body. The activation of Nrf2 increases glucose uptake in fibroblasts [144]. In contrast, interference in the supply of glucose has been shown to inhibit the detoxification pathways of reactive species mediated by Nrf2. In these pathways, we find the phosphate pentoses of the biosynthesis of nucleotides, the biosynthesis of serine, the glucuronidation pathway, and the tricarboxylic acids cycle. It has been determined that the induction of Nrf2 in SkM reduces glucose levels under feeding conditions and during the glucose intolerance test, and it suppresses weight gain in an animal model of induction to obesity [145]. In 2016, these same authors evaluated the impact that Nrf2 exerts on the metabolism of glucose by means of a model of SkM-Keap1 Knock Out (Keap1MuKO) mice and animals that were induced to express abundant Nrf2 in SkM, and in this manner, to examine the expression of the target genes of that tissue. It was found that Nrf2 disrupts the activity of various enzymes implicated in the hemostasis of glucose, specifically in the glycogen branching enzyme (GBE) and in subunit α of the phosphorylase b kinase (PhKα), reducing the amount of muscular glycogen, resulting in better glucose tolerance, thus regulating the metabolism of glycogenin in muscle and the liver [146].

Exercise demands the efficient coordination of the metabolic pathways to measure the supply of timely energy in all of the cells of the organism, predominantly, in those of the skeletal muscle but also of the organs and systems that undergo physiological adaptations under work conditions. According to this reasoning, it is indispensable to ascertain the elements that intervene in order for the totality of the processes to be carried out in optimal fashion. As previously mentioned, the mitochondria is the superior cellular producer organelle, fundamentally, in sports of prolonged durations and that predominantly involve the aerobic energy system. The potential of the mitochondrial membrane (Δψ_m_) is an indicator of the health of the mitochondria, and this is, to a large degree, determined by the electron transport chain and its elements. Some studies on the mitochondrial proteome point out that Nrf2 regulates the expression of certain mitochondrial proteins, such as subunit α of ATPsynthase [147]. In addition, Nrf2 exerts an influence on the effectiveness-of-function of mitochondrial complexes I and II for the consumption of oxygen, the production of ATP, the production of Kreb’s cycle substrates, and the equilibrium of the NADH redox index that intervenes in the mitochondrial function [74]. Thus, the latter is that in which, on the activation of Nrf2, the oxidative phosphorylation is more effective, there is more energy available for physical work along the exercise. The most interesting part of this is that Nrf2, upon being activated during exercise, connects the different antioxidant, energy, and detoxifying pathways in a complex communication network, with biofeedback to each other. For example, Nrf2 exerts an influence on the expression of enzymes such as ME1, IDH1, G6PD, PGD, and NADPH producers, utilizing the conversion of oxidized into reduced glutathione. It also participates in lipoxidation, favoring the flow of FADH to UbQ and to complex III in the electron transport chain. Additionally, it provides substrates for respiration and oxidative phosphorylation [74,148].

## 7. Clinical Implications of Therole of Nrf2 in Physical Exercise

As it has been mentioned, the transcriptional factor Nrf2 plays a crucial role in the maintenance of homeostasis, particularly in situations where OS elevation is implicated, due to its ability to induce the activity of cytoprotective elements including antioxidant enzymatic and non-enzymatic systems, detoxifying enzymes, and xenobiotics elimination. Moreover, Nrf2 functions as a regulator of several cellular processes such as proliferation, differentiation, and inflammatory response. In this sense, the understanding of these multiple processes, and at the same time, the cross-linking relationship between other elements facilitates the study of the molecular basis deeply related to many pathologies including diabetes, cardiovascular and neurodegenerative diseases and cancer. Many studies have also been carried out to explore the diverse forms of how Nrf2 can be expressed or inhibited [136].

One of the elements that has proved to induce Nrf2 expression is the increases of OS induced by physical exercise, which is responsible to produce acute and chronic physiological responses, resulting in different health benefits. Nowadays, formal exercise protocol design is one of the strategies to prevent or treat neurodegenerative disorders such as Alzheimer and Parkinson diseases or dementia. This is most likely due to the effect on neuronal plasticity, the increase of neurotrophic factors, anti-inflammatory cytokines and the reduced pro-inflammatory cytokines responsible for promoting neurodegenerative processes. The animal models have provided an understanding of nervous system dysfunctions [149].

The multi-organic, anti-inflammatory and antioxidant effect that physical exercise exerts on adipose and muscular tissue and the cardiovascular system to modulate the immune response has been described extensively, such as the expression of redox-sensible transcription factors and shock-heat protein synthesis [150]. It is important to understand the manner in which exercise can regulate the cytoprotective response mediated by Nrf2 as this would be a way to design specific training protocols to treat pathological events more effectively, and may make the patient willing to be responsible for their own health.

## 8. Conclusions

It is clear that the manner in which living beings evolved conferred on them the capacity to adapt to the environment, and the protection systems developed with the main aim of resisting injuries incurred by the constant changes. Although the Nrf2-mediated antioxidant protector system complex is not yet fully understood, it is known that it is efficient in the face of the response to stimuli such as exercise. The movement generated by any form of exercise implies the activation of energy-demanding cellular machinery. During this process, the production of electrophilic molecules is necessary to accelerate the signaling pathways that induce cellular changes and adaptations. There is evidence that provides the impact of different exercise modalities on the adaptative antioxidant and metabolic responses that induce hormesis; in other words, physical exercise is a way in which it is possible to influence the organism to become more resistant and for it to adapt in an increasingly efficient manner to environmental aggressions. However, it is imperative to study the effects of the training volume, frequency, intensity, and modality to be able to obtain more precise prescriptions, individualizing at every moment the specific conditions of each subject. It is a fact that, regardless of whether it is high-performance or recreational, exercise must be part of the lifestyle of every individual regardless of age, sex, or physical condition because the benefits go beyond physiological and molecular changes, and are manifested in different areas, including physical, emotional, mental, and economic health. In conclusion, more studies are necessary that sustain the molecular basis of the effect that physical exercise can have on the health of individuals.

## Figures and Tables

**Table 1:** Antioxidant cytoprotector system directed by Nrf2 [25,26,27,28,29,30,31].

Pathway/Enzimatic System	Activity
Glutathione synthesis and regeneration:	
-GCL: GCLM/GCLC complex	Carrying out glutathione synthesis forms glutamate and cysteine
-GPx	Detoxification of H_2_O_2_
-GR	Reduction of GSSG to GSH
-XCT	Transports cysteine to the cell to be reduced to cysteine from GSH
Phase-II detoxifying enzymes:	
-HO-1	Degradation of the heme group gives rise to biliverdin, free iron, and carbon monoxide
-UGT	Glucoronidation: conjugation of glucuronic acid
-SULT	Sulfonation: the addition of sulfuryl groups donated by 3′-PhosphoAdenosine-5′-PhosphoSulfate (PAPS) to hydroxyl or amine groups
Expression of NADPH- producing enzymes:	
-G6PD	Synthesis of NADPH in the PPP pathwa
-IDH	Synthesis of NADPH in the conversion ofisocytrate into α-ketoglutarate in the KC
-ME1	Synthesis of NADHP in the conversion of pyruvate into malate in the KC
Expression of Thioredoxins:	
-TXN1	Their two active cysteine residues can be oxidized for reducing the oxidized thiols of proteins
-TXNRD1	NADPH-dependent can reduce oxidized TXN
Detoxification of quinones:	
-NQO1	These compete with the CYP 450 reductases and convert quinones into more stable molecules (quinoles)
-AKR	
GCL: glutamate-cysteine ligase	SULT´s: sulfotransferases
GCLM: glutamate-cysteine ligase modifier subunit	G6PD: glucose-6-phosphate dehydrogenase
GCLC: glutamate-cysteine ligase catalytic subunit	PPP: pentose phosphate pathway
GST: glutathione S-transferases	NADPH: nicotinamine dinucleotide phophate
GR: glutathione reductase	IDH: isocitrate dehydrogenase
XCT: cystine/glutamate transporter	ME1: malic enzyme 1
GSSG: oxidized glutathione	KC: Kreb’s cycle
GSH: reducedglutathione	TXN1: thioredoxine 1
GPx: glutathioneperoxidases	TXNRD1: thioredoxine reductase 1
HO-1: heme oxygenase-1	NQO1: N-quinone oxido reductase 1
UGT: UDP-glucuronosyltransferases	AKR: aldo-ketoreductase
KC: Kreb’s cycle	

**Table 2:** Studies that have demonstrated the induction of Nrf2 and the antioxidant cytoprotector system due to the effect of physical exercise and bioactive compounds.

Model	Training Protocol	Objective	Results	Reference
Male mice Nrf2, WT, and KO aged 3 and 12 months	Free run on wheel for 6–8 weeks. Estimation of revolutions in 24 h and converted into distance (km)	Estimate the role of Nrf2 in biogenesis and mitochondrial content of SkM and the physical performance	Without difference in mitochondrial content ↓mitochondrial respiration, in KO mice: ↑ROS IMF ↓performance, ↑ fatigue. In WT ↑ COX. ET normalized ROS, performance, and respiration in KO	Crilly et al. [117]
Male mice 13 weeks of age Nrf2^+/+^ and Nrf2^−/−^	AT (5–10 m/min) 3 days prior to administration with SFN ExT on treadmill 5 m/min up to 28 m/min increase every 3 min	Evaluate performance, markers of damage, and OS ExT low conditions of ExT in mice administered SFN pre-treatment	↑distance covered by Nrf2^+/+^ SFN, ↓ markers of damage in Nrf2^+/+^ SFN after the SFN test → protection against muscle damage, regulation of Nrf2, and the antioxidant response. ↓ fatigue due to ↓of OS causing ExT	Oh et al. [118]
Young males aged 25 ± 1 years	HIIT Cycling protocol of 30 min Sample taking of blood before and after the session	Determine whether HIIT exercise can more efficiently evaluate Nrf2 than MET in humans	↑ Nrf2 in HIIT vs. MET ↑ GR activity and response ↑ 8-isoprostanes	Done et al. [119]
Male Sprague–Dawley rats aged 20–22 weeks	Exhaustive swimming every day for 3 weeks. After each session, the animals received 20–75 mg of LN or 100 mg of AA	Determine the effect of supplementation with LN on the diminution of fatigue and the modulation of the Nrf2/ARE pathway in a forced swimming model in rats	LN ↑performance resistance exercise normalized metabolic markers. ↓LA and LDH ↑capacity of resistance to the exercise ↑activity of antioxidant enzymes and antioxidant capacity ↓ TNFα, IL-1β, and IL-6 ↑ IL-10 anti-inflammatory in SkM and in liver	Duan et al. [120]
Male aged mice ICR/CD-1	AE at different durations (45, 90, 120, or 150 min)	Evaluate effect of AE on the Ref1/Nrf2 pathway, association with H_2_O_2_ and EAS	AE ↑ OS by the Ref1/Nrf2 pathway in time-dependent fashion in linear correlation of the content of H_2_O_2_ and the expression of Ref1/Nrf2. ↑GSH and ↑ activity of MnSOD. CuZnSOD not modified	Wang et al. [121]
Male mice C57/BL6/SJ aged 15–30 weeks Nrf2^+/+^ and Nrf2^−/−^	AT 5 days prior to the study, 5 min (0–9 m/min) 0 degrees of inclination ET included 30–60 min of treadmill running at 10–15 m min^−1^ at 10% inclination, 4–5 days per week. AE consisted of 1 h of treadmill running at 12 m min, at a 10° inclination	Determine the role of NFE2L2 in AE mitochondrial biogenesis and antioxidant response	ROS and NO regulate the expression of NFE2L2 in SkM cells ↓ NFE2L2 →↓ tolerance to exercise, mitochondrial density, and low SOD activity ↓of markers of mitochondrial biogenesis, citrate synthase, and mtDNA AE, NO, and H_2_O_2_→increase of NRF-1 and mtTFA was dependent on NFE2L2	Merry et al. [122]
Male mice aged 20 months Nrf2^+/+^ and Nrf2^−/−^	Test of previous resistance ability; 1 week of treadmill running for 10 min; 15–22 m/min; 0–12% inclination. HIES treadmill running for 6 weeks at 20–25 m/min; 12% inclination for 60 min per day	Determine the role of Nrf2 under stress by HIES in atrial cardiomyocyte hypertrophic changes	HIES →↑ markers of the gene expression of hypertrophy of cardiomyocytes (Anf, Bnf, and β-Mhc) in mice Nrf2^−/−^ ↓Gclc, Gsr, and Gstμ, levels of protein of NQO1, Cat, GPX1, GSH in Nrf2^−/−^ after HIES ↑expression of LC3 and ATG7 ↑ ubiquitination of ATG7 →↑ OS	Kumar et al. [123]
Male Wistar rats aged 8 weeks	1 week of adaptation CE: 25 m/min, 45 min/day, 5 days per week during 6 weeks Supplementation of Coenzyme Q10 at doses of 300 mg/kg	Investigate the effect of Coenzyme Q10 or ubiquinone on NFκB, IκB, Nrf2, and HO-1 after CET after 6 weeks of training in sedentary and active rats.	↓Significant NFκB in muscle, liver, and heart in the group that received Q10 post-training vs. sedentary group. ↑ Nrf2 and HO-1 in muscle, liver, and heart of the active group. ↓plasma triglycerides Without changes in metabolites related with CHO and proteins	Pala et al. [124]
Male mice Nrf2^+/+^ and Nrf2^−/−^ aged 2 months	AE on treadmill; 60 min/day, 14 m/min, 10% inclination, for 2 days.	Determine the impact of the AE in the activation of the Nrf2/ARE pathway and of the EAS system in mouse heart	↑activation of the Ref1/Nrf2 pathway and of the EAS pathway in mice Nrf2^+/+.^ ↑ OS and ↓ EAS (Cat, NQO1, GCS, GSR, GPx-1, G6PD, GSH) in Nrf2^−/−^ ↑activation of the Ref1/Nrf2 pathway and of the EAS pathway in Nrf2^+/+^ in old mice vs. Nrf2 AE protects against OS in cardiac muscle in mice	Muthusamy et al. [125]
Male mice C57/Bl6/SJ Young (aged ~2 months) and old (aged ≥23 months) Nrf2^+/+^ and Nrf2^−/−^	EES: 2 consecutive days on treadmill 90 min/day; 20 m/min; 12% inclination MET: 50 min/day; 10 m/min; 7% inclination min/day for 6 weeks. The protocol included 5 min of ramping at 5 m/min, the speed Increased to 10 m/min/45 min	Evaluate the regulation of Nrf2 depending on age, the antioxidant mechanisms, and redox equilibrium in mouse cardiac muscle Antioxidants under EES and MET conditions	↑susceptibility in old mice by OS produced by EES Proteins of the ARE antioxidant system ↑ in young mice, with respect to the old mice. ↓ Cat, NQO1 young mice Nrf2^−/−^, in old mice ↓G6PD, NQO1, cat, HO-1, and GPX1 MET: ↑Nrf2 nuclear and EAS in heart of old mice close to the levels of the young mice	Gounder et al. [34]

Abbreviations. Sol: soleus; EDL: extensor digitorum longus; SkM: skeletal muscle; WT: Wilde type; KO: Knock out; IMF: intramiofibrilar; COX: ciclooxiganase; AT: adaptation training; ExT: exercise training; SFN: sulforafano; OS: oxidative stress; ROS: reactive oxygen species; LN: luteolin-6-C-neohesperidoside; ascorbic acid; LA: lactic acid; TNFα: tumor necrosis factor-α; SkM: skeletal muscle; IL-1β: interleukin-1β; IL-6: interleukin-6; IL-10: interleukin-10; NFE2L2: nuclear factor erythroid-derived 2-like 2; mtTFA: mitocondrial transcription factor; NO: nitric oxide; H_2_O_2_: hydrogen peroxide; AE: acute training; HIES: high intensity exercise; CE: chronic exercise; HIIT: high-intensity interval training; EAS: endogen antioxidant system; MnSOD: manganese superoxide dismutase; CuZnSOD: cupper zinc superoxide dismutase; GSH: reduced glutathione; Cat: catalase NQO1; N-quinone oxido reductase-1; GCS: GSR, GPx-1: glutathione peroxidasa-1; G6PD: glycerladehide 6 phosphate deshydrogenase; GST: glutathione S-transferase; HO-1: heme oxigenase-1; Q_10_: coenzima Q_10_; CHO’s: carbohydrates; EES: exhaustive exercise; MET: moderate exercise training.
